# Design of Dust-Filtering Algorithms for LiDAR Sensors Using Intensity and Range Information in Off-Road Vehicles †

**DOI:** 10.3390/s22114051

**Published:** 2022-05-27

**Authors:** Ali Afzalaghaeinaeini, Jaho Seo, Dongwook Lee, Hanmin Lee

**Affiliations:** 1 Department of Automotive and Mechatronics Engineering, Ontario Tech University, Oshawa, ON L1G 0C5, Canada; ali.afsalaghaeinaeini@ontariotechu.net; 2 Department of Smart Industrial Machine Technologies, Korean Institute of Machinery & Materials, Daejeon 34103, Korea; lego0410@kimm.re.kr (D.L.); hmlee@kimm.re.kr (H.L.)

**Keywords:** LiDAR, filtering, algorithm, LIOR, LIDROR, de-dusting

## Abstract

Although the LiDAR sensor provides high-resolution point cloud data, its performance degrades when exposed to dust environments, which may cause a failure in perception for robotics applications. To address this issue, our study designed an intensity-based filter that can remove dust particles from LiDAR data in two steps. In the first step, it identifies potential points that are likely to be dust by using intensity information. The second step involves analyzing the point density around selected points and removing them if they do not meet the threshold criterion. To test the proposed filter, we collected experimental data sets under the existence of dust and manually labeled them. Using these data, the de-dusting performance of the designed filter was evaluated and compared to several types of conventional filters. The proposed filter outperforms the conventional ones in achieving the best performance with the highest *F1* score and removing dust without sacrificing the original surrounding data.

## 1. Introduction

Light detection and ranging (LiDAR) is a powerful sensing technology that can create a high-resolution map of an environment. For example, a prestigious LiDAR sensor such as VLP-16 can generate up to 600,000 points per second in a range of 100 m with an accuracy of 3 cm. Due to this merit, the LiDAR sensor has a wide range of applications in mobile robotics such as object detection [1,2], localization [3,4], and mapping [5,6]. However, the performance of LiDAR sensors is systematically affected when exposed to harsh environmental conditions such as dust [7] because, in contrast to Radar, the majority of commercial LiDAR sensors work around 900 nm wavelength, making them capable of sensing airborne particles. In such a situation, LiDAR sensors may not successfully distinguish between data coming from dust clouds and those from non-dust clouds. For example, Boss, a winner of the DARPA urban challenge competition, suffered from the same problem of falsely detecting dust as an object during the competition [8].

In the literature, there have been two major approaches used to address the problem of de-dusting. The first method is based on the data fusion from multiple sensors, including a camera, LiDAR, and radar. This approach takes advantage of a radar sensor that is less sensitive to dusty weather conditions, unlike LiDAR and cameras, even though it does not provide a high-quality map. For instance, ref. [9] used the depth fusion model to detect dust points that employed the discrepancy between LiDAR and radar.

The second approach is to exploit artificial-intelligence (AI) techniques such as machine learning and deep learning to classify point clouds into dust and non-dust points. For example, the authors in [10] identified dust point clouds using both machine-learning (ML) methods and custom neural networks. In this study, a 3D map was converted into 3D occupancy grids, and then meaningful information was extracted from the occupied voxels to train ML-based classifiers, i.e., random forest (RF) [11] and support vector machine (SVM) [12]. As input features for classification, the authors selected the mean and standard deviation of the intensity values of the points contained in each voxel as well as slope and roughness that can be obtained by applying principal component analysis (PCA) to the points inside the voxel [10]. The same voxel-based approach was chosen by [13] for fog classification. They used the SVM and *k*-nearest neighbors (KNN) algorithms as classifiers in which geometrical features and intensity were considered as inputs.

As another dust-filtering approach, a neural network classifier, was selected using advanced deep-learning methods [14]. Compared to [10], this study considered both point- and voxel-based classification. To improve its performance, they tested several input features for the classifier to find the best one for dust removal. These features are geometry, intensity as well as multi-echo information coming from the LiDAR sensor. Geometry and multi-echo features proved to be the most effective features for point-based deep-learning methods, while adding intensity information to these features brought a better solution for voxel-based deep-learning methods. Deep-learning methods were also applied to other adverse weather conditions. For example, the authors in [15] employed a CNN-based architecture named WeatherNet to filter out fog and rain noises in LiDAR point-cloud data. This method can segment point clouds using distance and intensity as input features. However, the aforementioned AI de-dusting methods have the following limitations [16]:

1. The first challenge comes from the size of the data. To collect data from environments using a LiDAR sensor, millions of points are needed, resulting in storage difficulties.
2. The large number of data sets required for training leads to high computation costs and training time.
3. The performance of this method is significantly dependent on the training data. In some particular situations where the AI model has not been trained, it may make a wrong decision.
4. In particular, in the deep-learning method, the model architecture must be insensitive to the detection distance and rotation of a sensor in order to maintain the filtering performance when the sensor (or robot equipped with the sensor) moves.

To overcome the limitations of the aforementioned de-dusting filters, the study of [17] presented an intensity-based filter for dust removal by taking advantage of LIOR (low-intensity outlier removal) filtering [18]. This paper evaluated the validity of the LIOR filtering method under different test conditions and identified the shortcomings. One limitation of the LIOR filter is that it deletes nearly all the points selected from the first step, which are apart from the sensor beyond a certain distance. Furthermore, selecting a low threshold on this filter causes some dust to remain after filtering, whereas increasing a threshold may result in the filtering process not being able to remove low-intensity objects completely in the second step.

To deal with the above problems, this paper proposes a new intensity-based algorithm for LiDAR sensors that can improve both dust filtering’s accuracy and robustness to the inherited sparsity of a LiDAR point cloud as distance increases. This improvement was achieved by redefining the second step of filtering to address the sparsity issue and enhance the capability of saving important environmental information. The proposed solution was experimentally evaluated using datasets collected by varying the LiDAR dust cloud and LiDAR target distances to represent various outdoor scenarios. The dataset was then manually labeled based on prior knowledge about the experimental scene. Using the labeled dataset, we tested the performance of the designed filters against the existing filters that were originally developed to remove noises for LiDAR sensors under adverse weather conditions such as snow [19,20,21] but were designed for de-dusting in this study. The considered existing filtering methods include statistical outlier removal (SOR) filter [19,21], radius outlier removal filter (ROR) [20], and dynamic radius outlier removal filter (DROR) [21].

The main contributions of our work can be summarized as follows.

- To the best of our knowledge, the proposed method is the first attempt to develop dust-filtering algorithms using non-AI techniques that take advantage of the inherent characteristics (intensity value) of dust point-cloud data.
- The proposed method can overcome the inherent problems of AI methods applied to dust filtering that require a large number of data sets for training and therefore lead to high computation costs and training time.
- This study provides an in-depth and comprehensive discussion of various design methodologies with SOR, ROR, DROR, LIOR, and LIDROR. Therefore, it can offer practical recommendations on which is the most suitable method through a comparative analysis.

The remainder of this paper is divided into the following sections. Section 2 provides a theoretical background on the existing filtering methods. In Section 3 and Section 4, the research methodologies and results of filtering evaluation are presented, respectively. The Section 5 discusses concluding remarks and future work.

## 2. Theoretical Background

In this section, we present the operational principles of several conventional de-noise filters for LiDAR point clouds that have been used to improve detection quality under harsh weather conditions.

### 2.1. SOR Filter

The SOR filter aims to remove the sparse outliers caused by measurement error [19,21]. To do so, it iterates through each point and then computes the average distances $d_{i}$ of *k*-nearest points to that point, where k represents an integer parameter of the filter that can be selected based on how many neighbor points are wanted to be analyzed [19]. As another key variable, the threshold value T can be defined as shown in Equation (1).

(1) T=μ±β×σ

where μ and σ are the mean and standard deviation of the average distances $d_{i}$, and β is a constant multiplier. This filter eliminates all points whose average distances fall outside the threshold interval. The performance of the SOR filter depends on the right selection of β and k.

### 2.2. ROR Filter

The ROR filter [20,21] removes isolated outliers from point clouds by iterating through each point and counting the number of points located within a sphere with a center of that point and search radius, R. It uses the k-d tree algorithm [22] to search for a point inside a sphere. If the number of points is less than the minimum acceptable number of points N, it is removed as an outlier, otherwise it is saved as an inlier. The parameters N and R can be varied to find an optimum solution for ROR filtering.

### 2.3. DROR Filter

In [21], the ROR and SOR filters were chosen for the first time to test their de-snowing abilities. This study found that the SOR was able to remove the majority of snow points, but failed to remove densely grouped snow points. Furthermore, although the ROR filter showed a better performance for de-snowing in general, it excluded all important information from the environment that was farther away than 18 m from a LiDAR sensor. This is because LiDAR points clouds become sparser as the distance from the sensor increases, while a search radius in the ROR filter remains constant.

To solve this problem, the study developed a DROR filter in which the search radius changes proportionally to the distance from the LiDAR sensor, as shown in Equation (2).

(2) $$R_{dynamic}=\phi \times \alpha \times \sqrt[{2}]{x^{2}+y^{2}}$$

where ϕ is a constant multiplier, α is the angular resolution of the LiDAR sensor, and [x,y] are the Cartesian coordinates of the point. The pseudocode for this filter is presented in Algorithm 1. The dynamic radius in Equation (2) enables rich data from the surroundings to be preserved while removing snow particles. To avoid a very small search radius for points near the LiDAR sensor, search radii less than the minimum search radius were set equal to the minimum search radius in the study.
**Algorithm 1** DROR filter1:**FOR** (Each point in the point cloud)2:     Search radius $\leftarrow \sqrt{x_{p}^{2}+y_{p}^{2}}$3:     **IF** (search radius < minimus search radius)4:        search radius = minimum search radius5:     **ELSE**6:        Search radius $\leftarrow \phi \times \alpha \times \sqrt{x_{p}^{2}+y_{p}^{2}}$7:     **ENDIF**8:        *n* ← Find number of points inside search radius9:        **IF** (*n* < threshold point)10:            Outliers ← point11:        **ELSE**12:            Inliers ← point13:     **ENDIF**14:**ENDFOR**

### 2.4. LIOR Filter

The methods outlined above rely on only geometry information from a LiDAR sensor for de-noising. An alternative approach, ref. [18], used the intensity information from LiDAR’s 3D point clouds for de-snow filtering based on the finding that snow particles have a lower intensity value than other objects. By applying this principle, the study of [18] proposed the LIOR filter, which consists of two stages. The first stage involves iterating through each point and identifying the points whose intensity is less than a threshold intensity value ϵ. Selecting the right threshold is crucial to the successful operation of the LIOR filter.

In the second stage, the ROR filter is applied to the selected points that have been identified as candidate outliers in the first step. In this stage, all the parameters related to the ROR filter, including the minimum acceptable number of points and the search radius, play a pivotal role. Finally, those points determined as outliers in the second step are removed from the point cloud.

The above procedure is summarized in Algorithm 2. The main feature of this filter is to apply the ROR only to selected points. This allows the LIOR filter to achieve a higher speed than the DROR filter while maintaining the same high level of performance as the DROR filter in terms of removing snow particles [18].
**Algorithm 2** LIOR filter1:**FOR** (Each point in the point cloud)2:     **IF** (point intensity > threshold intensity)3:        Inliers ← point4:     **ELSE**5:        % SR is sesrch radius6:        *n* ← Find number of points inside SR7:        **IF** (*n* < threshold point)8:            Outliers ← point9:        **ELSE**10:            Inliers ← point11:        **ENDIF**12:     **ENDIF**13:**ENDFOR**

## 3. Project Methodology

To develop a de-dusting filter, we first gathered LiDAR datasets under a dust environment to investigate the characteristics of dust clouds. An analysis of the collected data shows that dust particles have a low-intensity value. Therefore, we concluded that an intensity-based filtering method such as the LIOR can be applied to dust removal. Finally, we designed new dust-filtering algorithms by applying the LIOR and further developing it. These algorithms were implemented in MATLAB using a PC with Intel Core i5-8250U CPU. The algorithms consist of three parts to be processed: gathering data from the LiDAR sensor, analyzing data/filtering dust, and visualizing data in MATLAB. The first and the last parts were implemented using LiDAR Toolbox in MATLAB. The remainder of this section explains data collection, data analysis method, and filter design in detail.

### 3.1. Gathering Dust Dataset for Filter Design

Several datasets containing LiDAR data are publicly available, including the popular KITTI dataset [23], the A*3D Dataset [24], the nuScence Dataset [25], the Oxford RobotCar Dataset [26], the Canadian Adverse Driving Conditions Dataset [27], and the Waymo open dataset [28], but none of them include dust datasets. The Marulan dataset [29] contains LiDAR data containing airborne particles such as dust and smoke. However, no intensity information is provided here, and only 2D LiDAR sensors were used in their experiments. Thus, in order to develop our proposed dust-filtering algorithms using a 3D LiDAR, new datasets containing dust had to be created.

**Figure 1:**
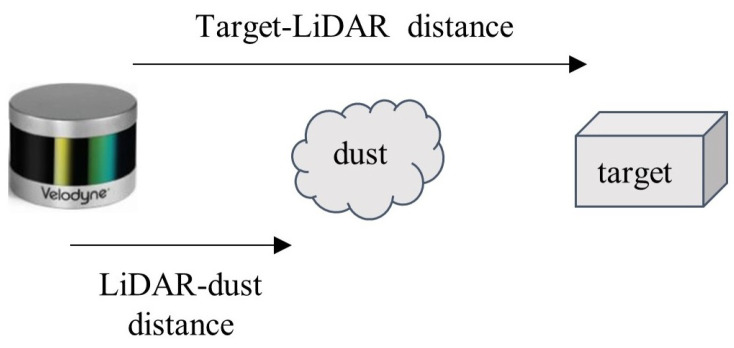
Design variables to generate experimental conditions.

According to [7], several parameters affect LiDAR measurements exposed to dust, which include the distance between a LiDAR and dust clouds, the distance between a LiDAR and a target, the dust cloud’s length, the dust density, the dust particle’s size, and the reflectivity and surface area of a target (reflected points). Among these parameters, the first two parameters (see Figure 1) were chosen as design variables to create different experimental conditions in this study. This is because the dust cloud’s length, the dust density, and the dust particle’s size are difficult to control; the reflectivity and surface area were not selected either for the sake of simplicity, as computing these quantities for every point in the point cloud complicates the problem. The distance between a LiDAR and the location of dust blowing was measured using a measuring tape in this study, and the target was placed at a predefined location described in Table 1.

**Figure 2:**
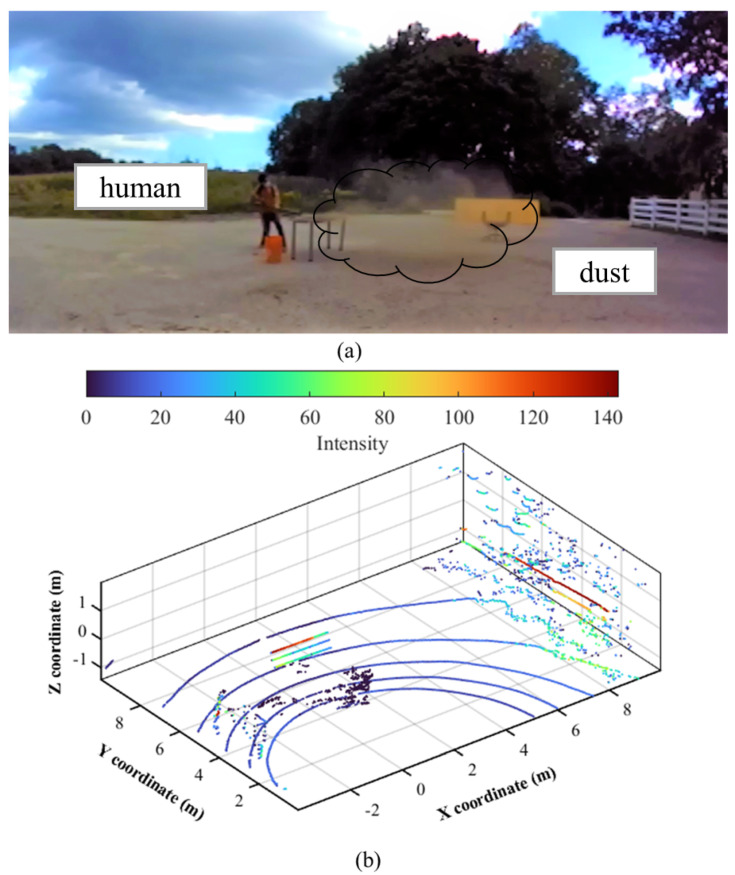
A scene of experimental data collection (**a**) and corresponding point cloud (**b**).

Therefore, we designed four different experimental conditions by varying these two variables as summarized in Table 1. Under these conditions, data were gathered with a VLP-16 [30] LiDAR sensor and a leaf blower on a clear day that was used to create dust particles. The experimental scene in Figure 2a includes a human, trees, and other background objects, as well as dust scattered by a blower.

### 3.2. Data Analysis Method

The obtained data was thoroughly examined to analyze the characteristics of measured point clouds. The primary characteristic of dust points is that their intensity ranges from 0 to 10, which is significantly lower than other objects. For instance, the point clouds were plotted according to their intensity values using the turbo colormap in Figure 2b where the dust point’s color is near black, equivalent to 0 of the intensity value. There were a few non-dust points as well, especially some ground points with low intensity (dark blue). In Figure 2b, dust noise (disturbance caused by dust) constitutes approximately 4.55 percent of the total point cloud that needed to be removed.

From the above observation, we can note that intensity is a viable criterion to classify or filter out dust point clouds. In the next step, the LIOR filter that requires the intensity information was applied to assess its capability and effectiveness in removing dust.

### 3.3. Optimizing LIOR for De-Dusting

As discussed in Section 2.4, the LIOR filter has three parameters: intensity threshold, search radius, and minimum acceptable number of points in the vicinity of a query point. Finding the right intensity threshold value is crucial for achieving a high-performance dust filter. Therefore, an analysis of the data was conducted to determine the appropriate threshold intensity.

**Figure 3:**
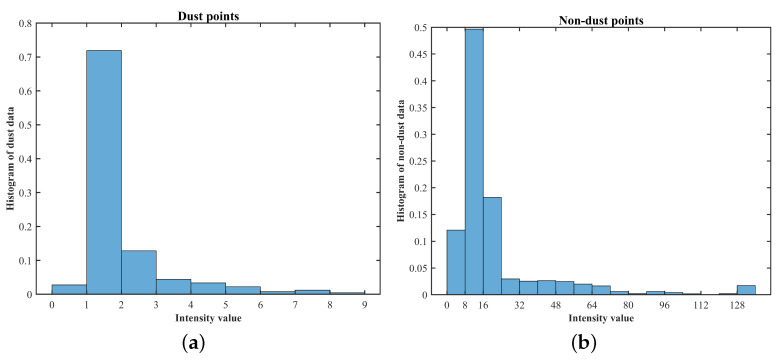
Histogram of VLP-16 LiDAR point clouds when exposed to dust: histogram of dust points as a percentage of total dust points (**a**) and histogram of non-dust points as a percentage of total non-dust points (**b**).

The histograms in Figure 3 illustrate the distribution of intensity values for dust and non-dust particles in Figure 2. In VLP-16, the intensity value varies as an integer ranging from 0 to 255. Specifically, the *x* axis presents an integer intensity interval while the *y* axis shows a fraction of the intensity data falling in each interval. For example, in Figure 3a, the *x* value of the 2nd bin is in the interval of [1,2) and its *y* value is about 71%. This means that 71% of dust points in Figure 3a have an intensity equal to 1. On the other hand, the majority of non-dust points, almost 88%, have an intensity greater than 8 (see Figure 3b).

**Figure 4:**
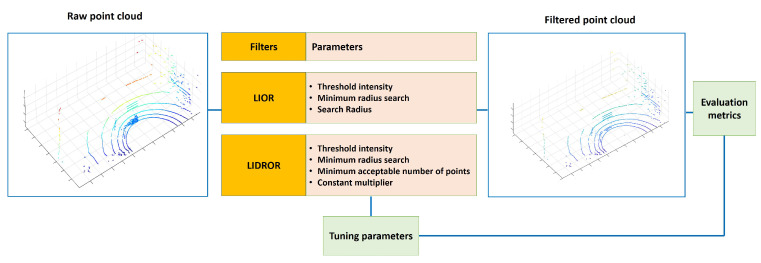
An illustration of how the tuning of LIOR and LIDROR were performed.

A high threshold increases the risk of removing low-intensity non-dust points. Therefore, there is a trade off between dust removal and preserving environmental information, and both have to be balanced. By considering both perspectives, 7 was selected as a threshold intensity in the study. As shown in Figure 4, the optimal values of the two remaining LIOR parameters, search radius and minimum acceptable number of points, in Table 2, were determined to provide the best filtering performance through trial and error using the data sets in Section 3.1.

### 3.4. Low-Intensity Dynamic Radius Outlier Removal (LIDROR)

To improve the LIOR filter to be more robust to distance variables, we devised a new filter named LIDROR. Specifically, the ROR filter was replaced by the DROR filter in the second stage of the LIOR filter to overcome the problem of the ROR’s filter by using a dynamic search radius (from line 5–9 of Algorithm 3). In this filter, the constant multiplier ϕ and the minimum acceptable number of points within the search radius are the parameters to be tuned for de-dusting. Based on observations of how these parameters affect the filtering performance and robustness in different dust scenarios, they were tuned accordingly.

The LIDROR filter also has the merit of allowing the threshold intensity to be set higher without sacrificing important non-dust information while maximizing dust removal. Through experiments, 8 was determined as a threshold intensity for this filter, which is higher than 7 for the LIOR filter. The finalized parameter values including the threshold intensity are summarized in Table 3.
**Algorithm 3** LIDROR filter1:**FOR** (Each point in the point cloud)2:     **IF** (point intensity > threshold intensity)3:        Inliers ← point4:     **ELSE**5:        **IF** ( search radius < minimum search radius)6:            search radius = minimum search radius7:        **ELSE**8:            Search radius $\leftarrow \phi \times \alpha \times \sqrt{x_{p}^{2}+y_{p}^{2}}$9:        **ENDIF**10:        *n* ← Find number of points inside SR11:        **IF** (*n* < threshold point)12:            Outliers ← point13:        **ELSE**14:            Inliers ← point15:        **ENDIF**16:     **ENDIF**17:**ENDFOR**

## 4. Results and Discussion

**Figure 5:**
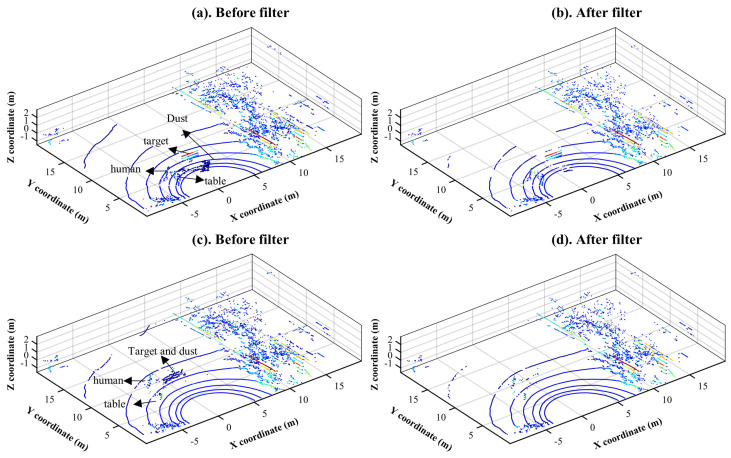
Experimental results after applying the developed LIOR de-dusting filters in two different scenarios: Point cloud map before filtering in case of experiment No. 1, first scenario (**a**); point-cloud map after LIOR filtering in case of experiment No. 1, first scenario (**b**); point-cloud map before filtering in case of experiment No. 2, second scenario (**c**); and point-cloud map after LIOR filtering in case of experiment No. 2, second scenario (**d**).

**Figure 6:**
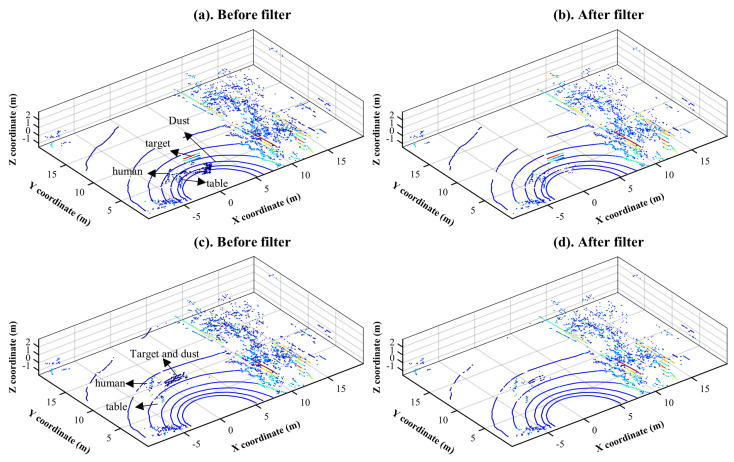
Experimental results after applying the developed LIDROR de-dusting filters in two different scenarios: point-cloud map before filtering in case of experiment No. 1, first scenario (**a**); point-cloud map after LIDROR filtering in case of experiment No. 1, first scenario (**b**); point-cloud map before filtering in case of experiment No. 3, second scenario (**c**); and point-cloud map after LIOR filtering in case of experiment No. 3, second scenario (**d**).

Figure 5 and Figure 6 show the results of dust removal with the designed filters, LIOR and LIDROR, in two different scenarios from Table 1. In the first scenario, dust clouds are located approximately 4 m from a LiDAR sensor, which is equivalent to experiment No. 1 in Table 1. They are located within 8 m in the second scenario (experiment No. 3 in Table 1).

Figure 5a,c present the point-cloud maps without filtering in the first and second test scenarios, respectively, and Figure 5c,d display the maps after applying the LIOR filter in each case. As shown in Figure 5b,d, the LIOR filter removed almost all dust points. Some of the non-dust points having a low-intensity value can also be saved in the second stage (ROR) of the LIOR filter; since non-dust points are dense and the ROR filter can save them, especially if they are not far away from the sensor. However, some low-intensity non-dust points from the environment were also eliminated. The distance from the LiDAR sensor to these eliminated points was approximately more than 15 m in both figures. As mentioned in the theoretical background Section 2.2, this is a drawback of the ROR filter, a part of the LIOR filter, which deletes almost all points that are far away from the sensor. Another limitation of this method is that it is difficult to choose a higher threshold intensity since it is likely to detect more low-intensity points from the environment in the first step and then increase the risk of removing these non-dust points from the environment using the ROR filter in the second step.

The proposed LIDROR filter was also tested as illustrated in Figure 6, using the same point clouds used for the evaluation of the LIOR filter. This filter can save low-intensity non-dust points at a long distance from the sensor. As it can be seen in Figure 6d, this filter can remove dust points while keeping the information of target points whose intensity return is low.

**Figure 7:**
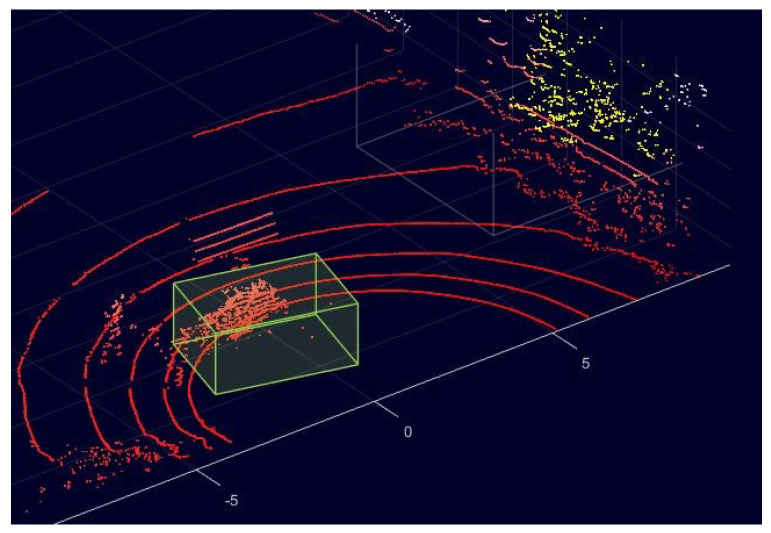
Labeling data in MATLAB LiDAR labeler app: The points inside a yellow cube are considered as dust.

To evaluate our proposed LIOR and LIDROR de-dusting filters, we manually labeled some of the collected data based on prior knowledge about the experimental scene. This work was carried out using the LiDAR labeler app in MATLAB [31], as illustrated in Figure 7, which enables us to draw a cuboid around the dust cloud and label it as dust. In the figure, the points inside the yellow cuboid are labeled as dust. Consequently, dust and non-dust point clouds are labelled 1 and 0, respectively. The metrics used for evaluating the filtering performance are accuracy, precision, recall, and *F1*-score, defined in Equations (3)–(6).

(3) $$\begin{array}{c} Accuracy=\frac{TP+TN}{N} \end{array}$$

(4) $$\begin{array}{c} Precision=\frac{TP}{TP+FP} \end{array}$$

(5) $$\begin{array}{c} Recall=\frac{TP}{TP+FN} \end{array}$$

(6) $$\begin{array}{c} F1\text{-}score=\frac{2}{\frac{1}{Recall}+\frac{1}{Precision}} \end{array}$$

where *TP* is the number of dust points that are removed correctly, *TN* is the number of non-dust points that are saved correctly, *FP* is the number of non-dust points that are removed as dust falsely, *FN* is the number of dust points that are preserved as non-dust falsely, and *N* is the total number of points inside the point cloud. A high precision score indicates a low *FP*, implying that the filter is effective at removing dust noise. On the other hand, a high recall score indicates a low *FN*, which means that the filter can effectively preserve environmental information.

These filters were then compared to the existing de-noising filters, SOR, ROR, and DROR whose parameters are summarized in Table 4. All the candidate filters were applied to the point clouds used in Figure 5a and Figure 6a, which correspond to experiments No. 1 and 3 in Table 1.

According to the evaluation results with the four metrics, as shown in Table 5, the SOR filter has the worst overall performance for removing dust noise that constitutes 4% of the total point cloud. The SOR filter, however, has a higher accuracy value than the ROR. As the SOR only considers the *k*-nearest points when removing outliers, this filter is ideal for removing noises that are isolated from others (i.e., removing sparse outliers). However, because the dust point cloud contains a very small number of isolated points, this filter is ineffective in removing dust.

On the other hand, the performance of the ROR filter for removing dust depends on the selected radius search, since a small radius search results in a loss of significant useful information about the environment. The ROR outperforms the SOR as it considers the density of neighbor points. The DROR filter delivers better results than the ROR and SOR by addressing the sparsity issue in the LiDAR points cloud. However, due to the same limitation as the ROR, choosing a smaller search radius than the current one cannot improve the de-noise (dust) performance.

The LIOR filter is comparable to the LIDROR in terms of removing dust. However, because the LIOR eliminates nearly all the non-dust points selected from the first step beyond a certain distance, it has a lower recall score than LIDROR due to the sparseness of a LiDAR point cloud at long range. Among the five filters, the LIDROR has the best performance across all metrics with an outstanding *F*1-score of 97.55%. In addition, it has the highest recall value (95.74%) and a precision value near 100%, indicating that this filter is not only able to maintain the environmental data, but also eliminate almost all of the dust from the point cloud.

Although LIDROR has a better *F1*-score than LIOR, it is computationally more expensive. The processing time for filtering takes around 0.383 and 0.412 s for the LIOR and LIDROR, respectively.

## 5. Conclusions

This paper aims to design noise-filtering algorithms that can remove dust from LiDAR sensory data for mobile machines in industrial sectors facing dust environments. To achieve the goal, we developed the intensity-based filter (LIDROR) based on an in-depth analysis of the properties of dust point clouds measured using a LiDAR sensor. To the best of our knowledge, the proposed method, along with our previously developed LIOR, are the first attempts to design a de-dust filter using non-AI techniques in this field.

To evaluate the developed de-dusting algorithms, four different metrics were used with the manually labeled data sets. The performance of the developed algorithms was compared with that of the SOR, ROR, and DROR filters that were previously applied for noise filtering in harsh weather conditions such as snow.

Evaluation results show that the proposed LIOR and LIDROR filters outperformed the conventional filters. Moreover, the LIDROR provides the most accurate and robust performance for dust removal with an *F*1-score of 97.55. It is expected from the results that our proposed filters can be used in applications such as mining and off-road machinery under harsh environmental conditions with dust. The intended future work is to implement the developed filters on a mobile platform and further test them under more various and dynamic scenarios (e.g., varying dust conditions such as dust density, including moving objects to detect, etc.). Finally, AI techniques will be applied to design different types of de-dust filters and their performance will be compared to that of non-AI filters proposed in this study.

## Figures and Tables

**Table 1:** Experimental conditions.

No. Experiment	LiDAR–Dust Cloud Distance	LiDAR–Target Distance
1	4	5
2	5	10
3	8	10
4	10	15

**Table 2:** LIOR final parameters.

LIOR Parameters	Value
Threshold intensity	7
Search radius (m)	0.044
Minimum acceptable number of points	6

**Table 3:** LIDROR final parameters.

LIDROR Parameters	Value
Threshold intensity	8
Minimum radius search (m)	0.044
Minimum acceptable number of points	5
Constant multiplier	0.011

**Table 4:** Parameters of the SOR, ROR, AND DROR filters.

Filter	Parameters	Value
SOR	*K*-nearest number	8
	Constant multiplier	0.1
ROR	Search radius	0.04
	Minimum acceptable number of points	3
DROR	Minimum search radius	0.04
	Minimum acceptable number of points	3
	Constant multiplier	

**Table 5:** Evaluation results.

Filters	Evaluation Metrics (%)			
	Accuracy (%)	Precision (%)	Recall (%)	*F*1-Score (%)
SOR	86.3	0.21	0.33	0.26
ROR	73.11	10.77	54.25	17.97
DROR	91.63	36.78	75.49	49.46
LIOR	89	99.27	89.87	94.24
LIDROR	95.46	99.44	95.74	97.55

## Data Availability

Not applicable.
